# Nectin-4 regulates cellular senescence-associated enlargement of cell size

**DOI:** 10.1038/s41598-023-48890-z

**Published:** 2023-12-07

**Authors:** Ryoko Katasho, Taiki Nagano, Tetsushi Iwasaki, Shinji Kamada

**Affiliations:** 1 Department of Biology, Graduate School of Science, Kobe University, 1-1 Rokkodai-Cho, Nada-Ku, Kobe, 657-8501 Japan; 2 Biosignal Research Center, Kobe University, 1-1 Rokkodai-Cho, Nada-Ku, Kobe, 657-8501 Japan

**Keywords:** Cell biology, Cell adhesion, Cell death, Cell signalling, Senescence

## Abstract

Cellular senescence is defined as irreversible growth arrest induced by various stress, such as DNA damage and oxidative stress. Senescent cells exhibit various characteristic morphological changes including enlarged morphology. In our recent study, we identified Nectin-4 to be upregulated in cellular senescence by comparative transcriptomic analysis. However, there are few reports on the relationship between Nectin-4 and senescence. Therefore, we analyzed the function of Nectin-4 in senescence and its biological significance. When overexpressed with Nectin-4, the cells exhibited the enlarged cell morphology closely resembling senescent cells. In addition, the cell size enlargement during DNA damage-induced senescence was suppressed by knockdown of Nectin-4, while there were no significant changes in senescence induction. These results suggest that Nectin-4 is not involved in the regulation of senescence itself but contributes to the senescence-associated cell size increase. Furthermore, the Nectin-4-dependent cell size increase was found to be mediated by Src family kinase (SFK)/PI3 kinase (PI3K)/Rac1 pathway. To explore the functional consequences of cell size enlargement, we analyzed cell survival in Nectin-4-depleted senescent cells. Single-cell tracking experiments revealed that Nectin-4 knockdown induced apoptosis in senescent cells, and there is a strong positive correlation between cell size and survival rate. These results collectively indicate that Nectin-4 plays a causative role in the senescence-associated cell size enlargement via SFK/PI3K/Rac1, which can contribute to survival of senescent cells.

## Introduction

Cellular senescence is defined as irreversible growth arrest induced by various stresses, such as DNA damage, oxidative stress, activated oncogenes, and telomere shortening^1–3^. Although senescence has been considered to be a potent tumor suppressor mechanism due to its ability to limit the proliferation of damaged cells, it has also become evident that accumulation of senescent cells in tissues can promote age-related pathologies, including tumor progression, Alzheimer’s disease, atherosclerosis, cardiovascular dysfunction, and so on, largely through the cell non-autonomous effects of the senescence-associated secretion of diverse pro-inflammatory cytokines and chemokines^4–9^. Senescent cells are known to exhibit a variety of distinctive morphological changes including flattened and enlarged morphology^10–13^. However, despite the early discovery of senescence-associated morphological changes in the 1960s, it has remained uncertain how this process is regulated in senescence.

Nectins (Nectin-1, -2, -3, and -4) comprise a family of calcium-independent immunoglobulin-like molecules that induce cell–cell adhesion through homophilic and heterophilic trans-interactions^14–17^. Besides cell adhesion, Nectins regulate various cellular functions, such as cell movement, polarization, survival, and differentiation by interacting with multiple types of proteins, including integrins, afadin, PARD3, PICK1, MUPP1, MPP3, zyxin, and willin. Northern blot analysis of normal adult human multiple tissues has shown that Nectin-1, and -2 are widely expressed in tissues, while Nectin-4 is mainly expressed in placenta and trachea^17^. However, we have recently identified *Nectin-4* (also known as *PVRL4*) to be upregulated preferentially in senescent cells by transcriptomics, and the *Nectin-4* upregulation is dependent on p53, a critical transcription factor required for the initiation and establishment of senescence^18–20^. It is still unclear whether Nectin-4 plays a role in the regulation of senescence, because in our previous study, enforced expression of Nectin-4 in osteosarcoma U2OS cells had no significant effect on senescence-associated β-galactosidase (SA-β-gal) staining^18^, an established marker of senescence^21, 22^.

Rac1 is a member of Rho family small GTPases that mediate a plethora of cellular functions such as regulation of cellular architecture, cell size, adhesion, polarity, motility, and so on^23, 24^. Rac1 is activated by guanine nucleotide exchange factors (GEFs) that promote the exchange of GDP for GTP molecules. The Rac1 activity is regulated by extracellular stimuli including various receptor tyrosine kinases. For example, epidermal growth factor receptor (EGFR) regulates Rac1 activity through the activation of phosphoinositide 3-kinases (PI3K) that in turn activates Tiam, a GEF for Rac1. Rac1 has been reported to increase cell size by enhancing the formation of filopodia^25, 26^. However, to date, the relationship between Rac1 and the senescence-associated morphological changes has yet to be described.

In this study, we investigated the relationship between Nectin-4 and senescence. We found that Nectin-4 is not involved in the regulation of senescence induction itself. However, Nectin-4 is shown to be responsible for the cell size enlargement associated with senescence. Furthermore, Src family kinase (SFK)-PI3K-Rac1 signaling is required for the Nectin-4-dependent cell size enlargement.

## Results

### Nectin-4 regulates senescence-associated cell size enlargement

**Figure 1:**
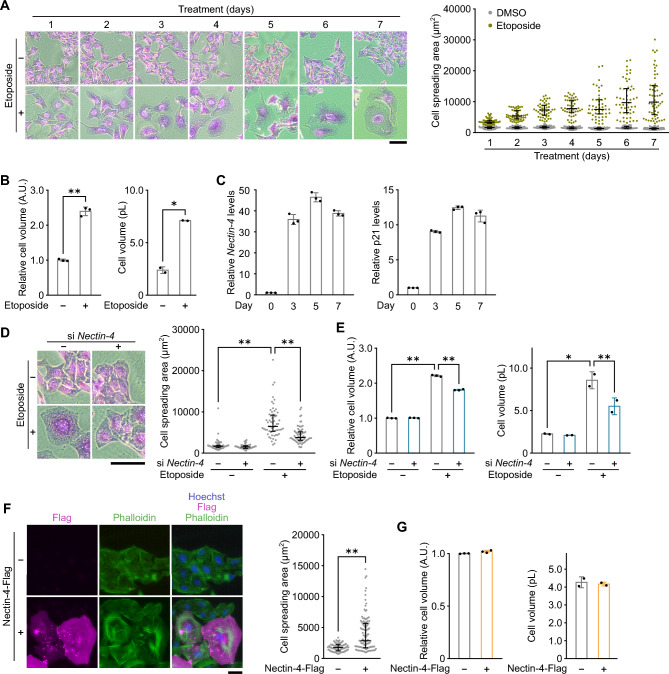
Nectin-4 regulates senescence-associated cell size enlargement in U2OS cells. (**A**) U2OS cells treated with 2 μM etoposide for the indicated times were stained with crystal violet, and cell area was measured by microscopic image analysis. Representative microscopic images (left panel) and dot plots of cell area between 5 and 95th percentiles (right panel) are shown. Upper and lower error bars (whiskers) and lines across the whiskers indicate the 75th and 25th percentiles and the median, respectively. Bar, 200 μm. (**B**) Left panel: U2OS cells treated with 2 μM etoposide for 3 days were subjected to cell volume measurement by flow cytometry. Cell volume data are median ± s.d. (n = 3 independent cultures). Right panel: U2OS cells treated with 2 μM etoposide for 7 days were subjected to cell volume measurement by Scepter cell counter. Cell volume data are mean ± s.d. (n = 2 independent cultures). (**C**) U2OS cells treated with 2 μM etoposide for the indicated times were subjected to qPCR analysis for *Nectin-4* and *p21*. Data are mean ± s.d. (n = 3 independent cultures). (**D**) U2OS cells transfected with siRNA for *Nectin-4* and treated with 2 μM etoposide as indicated for 3 days were stained with crystal violet (left panel) and subjected to cell area measurement by microscopic image analysis (right panel) as described in (**A**). Bar, 200 μm. (**E**) Left panel: U2OS cells transfected with siRNA for *Nectin-4* and treated with 2 μM etoposide as indicated for 3 days were subjected to cell volume measurement by flow cytometry. Cell volume data are median ± s.d. (n = 3 independent cultures). Right panel: U2OS cells transfected with siRNA for *Nectin-4* and treated with 2 μM etoposide as indicated for 7 days were subjected to cell volume measurement by Scepter cell counter. Cell volume data are mean ± s.d. (n = 2 independent cultures). (**F**) U2OS cells transfected with p3XFLAG-CMV-14-Nectin-4 were subjected to immunostaining with the anti-Flag antibody (magenta) and to staining with phalloidin (green) and Hoechst (blue), and cell area was measured by microscopic image analysis. Representative microscopic images (left panel) and dot plots of cell area between 5 and 95th percentiles (right panel) are shown as described in (**A**). Bar, 50 μm. (**G**) Left panel: U2OS cells were transfected with p3XFLAG-CMV-14-Nectin-4, and cell volume was measured by flow cytometry. Data are median ± s.d. (n = 3 independent cultures). Right panel: U2OS cells were transfected with p3XFLAG-CMV-14-Nectin-4, and cell volume was measured by Scepter cell counter. Cell volume data are mean ± s.d. (n = 2 independent cultures). Statistical significance is shown using the Student’s *t*-test analysis; **p < 0.01; *p < 0.05.

Senescent cells are known to show enlarged morphology^12, 13^. Actually, we observed that upon the treatment with etoposide, the cells were increased in cell size (cell spreading area) in a time-dependent manner (Fig. 1A). To investigate whether cell volume is also associated with the change of cell spreading area, U2OS cells treated with etoposide were analyzed by flow cytometer using forward scatter as a measure of cell volume and Scepter cell counter (Fig. 1B). As a result, cell volume was also increased in response to the etoposide treatment, consistent with the reported phenotype of senescent cells^27^. Comprehensive analyses have identified that Nectin-4 was upregulated in senescent cells induced by various stresses, such as DNA damage, telomere shortening, and oncogene^18, 28, 29^. Therefore, we confirmed whether the Nactin-4 expression was induced in human osteosarcoma U2OS cells treated with a sub-lethal dose of etoposide, which effectively induces senescence^18^. As shown in Fig. 1C, the *Nectin-4* mRNA expression was gradually upregulated in response to the etoposide treatment with a peak at 5 days, similar time course with the *p21* expression.

To investigate the function of Nectin-4 in cellular senescence, we analyzed the morphological changes of *Nectin-4* knockdown cells. The efficacy of *Nectin-4* knockdown was confirmed by quantitative PCR (qPCR), because we could not reproducibly detect the endogenous Nectin-4 protein by immunoblot analysis presumably due to its low expression at the basal level. *Nectin-4* expression upregulated by etoposide treatment was efficiently abolished by the treatment of siRNA targeting *Nectin-4* (Fig. S1A). The cell area and volume were measured after 3 days of etoposide treatment to eliminate the possible confounding effects of long-term culture on the cell morphology. siRNA-mediated knockdown of *Nectin-4* effectively impaired the senescence-associated increase in both cell area and volume (Fig. 1D,E), suggesting that Nectin-4 functions in the regulation of cell size in terms of both spreading area and volume. Next, we constructed an expression vector containing C-terminal Flag-tagged Nectin-4. After introduction of Nectin-4 into U2OS cells, Nectin-4 protein expression was validated by immunofluorescence and immunoblot analyses (Fig. S1B,C). Nectin-4 was found to be distributed throughout the plasma membrane (Fig. S1B), which is consistent with the known role of Nectin-4 as a plasma membrane adhesion molecule^15, 16^. Intriguingly, when overexpressed with Nectin-4, the cells exhibited an enlarged cell morphology closely resembling senescent cells, and this observation is accompanied by the characteristic increase in F-actin polymerization seen in senescent cells despite the absence of etoposide treatment (Fig. 1F, left panel). The cell spreading area was significantly increased by overexpression of Nectin-4, as determined by measurement of phalloidin stained area (Fig. 1F, right panel). These results raised the possibility that Nectin-4 contributed to the cell size increase associated with the senescence establishment. Ectopic expression of Nectin-4, however, resulted in only modest increase in cell volume (3%) (Fig. 1G), suggesting that Nectin-4 upregulation alone cannot explain the senescence-associated increase in cell volume.

### Nectin-4 is not involved in DNA damage-induced senescence itself

To investigate whether Nectin-4 directly regulates senescence-associated cell enlargement, or merely affects senescence induction itself, the extent of DNA damage-induced cellular senescence in the Nectin-4-overexpressed cells was determined by two widely used senescence markers, SA-β-gal staining^21, 22^ and loss of proliferative capacity judged by EdU incorporation assay. When U2OS cells were treated with a sub-lethal dose of etoposide, cellular senescence was effectively induced (Fig. S1D,E), which is consistent with previous results^18, 20, 30^. More importantly, overexpression of Nectin-4 did not affect both the SA-β-gal activity and proliferative capacity regardless of whether etoposide was present or not. Furthermore, knockdown of *Nectin-4* did not affect etoposide-induced senescence (Fig. S1F,G). These results suggested that Nectin-4 is not involved in the regulation of senescence induction itself, but rather can be related to another aspect of senescence.

### Nectin-4 regulates senescence-associated cell size enlargement in a cell type- or a stimulus-independent manner

**Figure 2:**
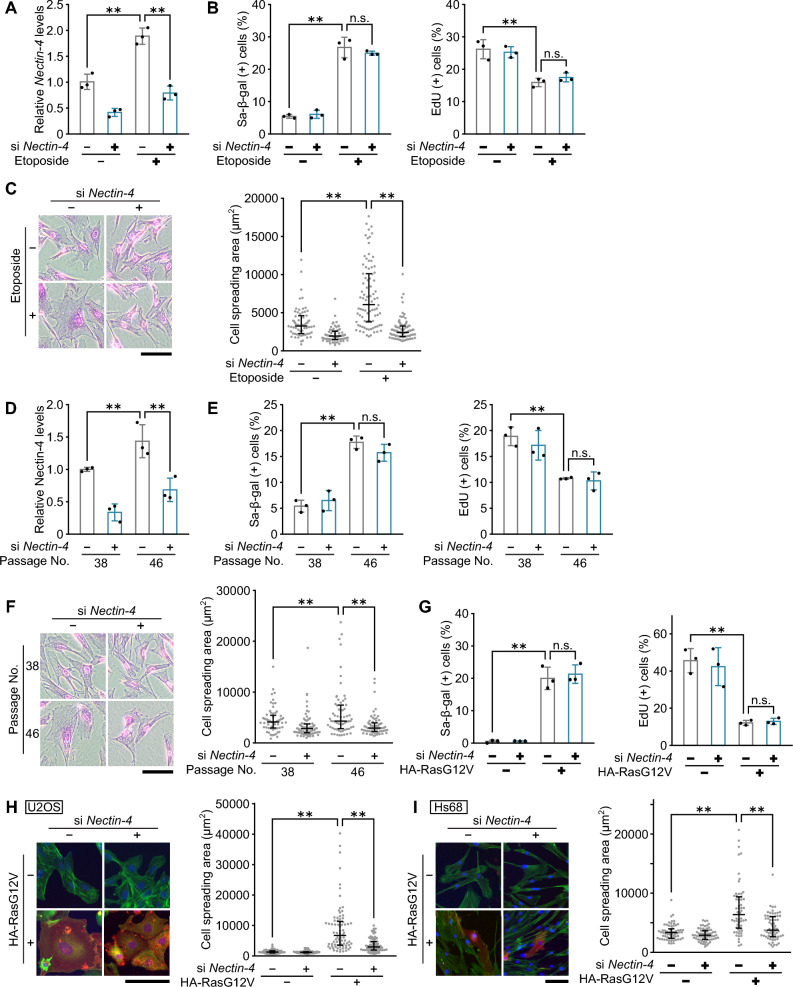
Nectin-4 regulates senescence-associated cell size enlargement in a cell type- or a stimulus-independent manner. (**A**-**C**) Hs68 cells transfected with siRNA for *Nectin-4* and treated with 0.5 μM etoposide as indicated for 3 days were subjected to qPCR analysis (**A**), SA-β-gal and EdU incorporation assays (**B**), and cell area measurement by microscopic image analysis after staining with crystal violet (**C**). (**B**) The percentage of SA-β-gal positive cells (left panel) and of EdU positive cells (right panel) are shown. (**C**) Representative microscopic images (left panel) and dot plots of cell area between 5 and 95th percentiles (right panel) are shown. Upper and lower error bars (whiskers) and lines across the whiskers indicate the 75th and 25th percentiles and the median, respectively. Bars, 200 μm. (**D**-**F**) Hs68 cells at passage numbers 38 (young) and 46 (early senescent) transfected with siRNA for *Nectin-4* as indicated were subjected to qPCR analysis (**D**), SA-β-gal and EdU incorporation assays (**E**), and cell area measurement by microscopic image analysis after staining with crystal violet (**F**). (**E**) The percentage of SA-β-gal positive cells (left panel) and of EdU positive cells (right panel) are shown. (**F**) Representative microscopic images (left panel) and dot plots of cell area between 5 and 95th percentiles (right panel) are shown as described in (**C**). Bars, 200 μm. The reduced-scale images are presented in Fig. S3. (**G**) U2OS cells were transfected with siRNA for *Nectin-4* and with pcDNA3-HA containing oncogenic RasG12V as indicated, and were selected with 800 μg/ml G418. After incubation for 5 days, the cells were subjected to SA-β-gal staining (left panel) and EdU incorporation assay (right panel). (**H**) U2OS cells were transfected with siRNA for *Nectin-4* and with pcDNA3-HA containing oncogenic RasG12V as indicated. After incubation for 5 days, the cells were stained with Hoechst (blue), phalloidin (green), and anti-HA antibody (red), respectively. Representative microscopic images (left panel) and dot plots of cell area between 5 and 95th percentiles (right panel) are shown as described in (**C**). Bar, 100 μm. The reduced-scale images are presented in Fig. S3. (**I**) Hs68 cells were transfected with siRNA for *Nectin-4* and with pcDNA3-HA containing oncogenic RasG12V as indicated. After incubation for 5 days, the cells were stained with Hoechst (blue), phalloidin (green), and anti-HA antibody (red), respectively. Representative microscopic images (left panel) and dot plots of cell area between 5 and 95th percentiles (right panel) are shown as described in (**C**). Bar, 100 μm. The reduced-scale images are presented in Fig. S3. Data are mean ± s.d. (n = 3 independent cultures). Statistical significance is shown using the Student’s *t*-test analysis; **p < 0.01; n.s., not significant (*p* > 0.05).

To test the generality of the relationship between Nectin-4 and the senescence-associated cell size enlargement, we conducted similar experiments using normal human fibroblast Hs68 cells. As is the case in U2OS cells, the etoposide-induced upregulation of *Nectin-4* was also observed in Hs68 cells, which was abolished by siRNA-mediated knockdown of *Nectin-4* (Fig. 2A). Furthermore, DNA damage-induced senescence was also induced in Hs68 cells by the treatment with the sub-lethal dose of etoposide, which was not affected by *Nectin-4* knockdown (Fig. 2B). Most importantly, knockdown of *Nectin-4* effectively suppressed the etoposide-induced increase in spreading area of Hs68 cells (Fig. 2C). In addition, when replicative senescence was induced by serial passage of Hs68 cells, *Nectin-4* expression was upregulated and its depletion impaired senescence-associated cell size increase but not senescence induction itself (Fig. 2D-F). Furthermore, cell spreading by oncogenic Ras-induced senescence of U2OS cells and Hs68 cells was also impaired by *Nectin-4* knockdown (Fig. 2G-I). These results indicate that Nectin-4 is generally involved in the regulation of senescence-associated morphological enlargement regardless of the stimuli or cell type.

### Nectin-4 induces cell size enlargement through SFK-PI3K pathway

**Figure 3:**
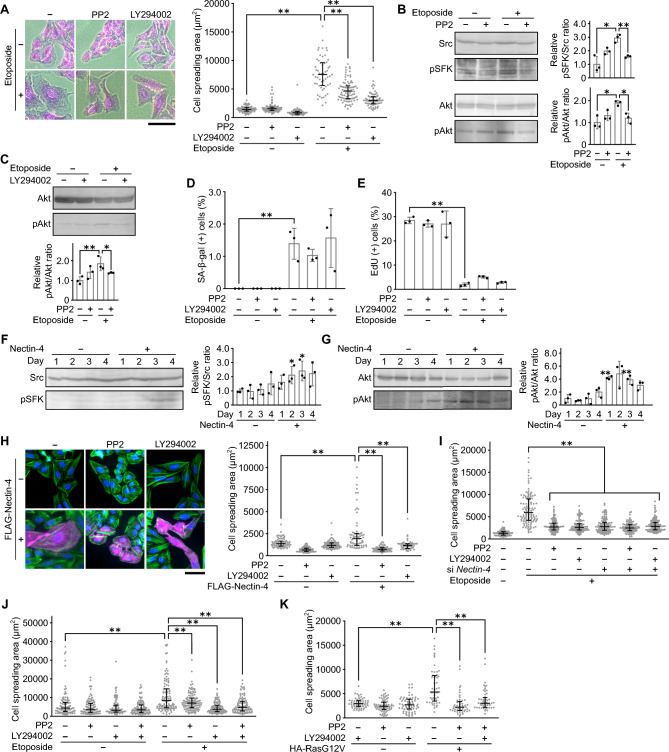
Nectin-4 induces cell size enlargement through SFK-PI3K pathway. (**A**) U2OS cells treated with 10 μM PP2 or 20 μM LY294002 in the presence of 2 μM etoposide as indicated for 3 days were subjected to cell area measurement by microscopic image analysis after staining with crystal violet. Representative microscopic images (left panel) and dot plots of cell area between 5 and 95th percentiles (right panel) are shown. Upper and lower error bars (whiskers) and lines across the whiskers indicate the 75th and 25th percentiles and the median, respectively. Bar, 200 μm. (**B**) U2OS cells treated with 10 μM PP2 in the presence of 2 μM etoposide as indicated for 3 days were subjected to immunoblot analysis. The phosphorylated protein levels of SFK and Akt normalized to their corresponding total protein levels were quantified using NIH ImageJ software. Representative immunoblot images from replicate experiments (n = 3, left panels) and the results of their statistical analysis (right panels) are shown. Original blots are presented in Fig. S4. (**C**) U2OS cells treated with 20 μM LY294002 in the presence of 2 μM etoposide as indicated for 3 days were subjected to immunoblot analysis. The phosphorylated protein levels of Akt normalized to their corresponding total protein levels were quantified using NIH ImageJ software. Representative immunoblot images from replicate experiments (n = 3, top panel) and the results of their statistical analysis (bottom panel) are shown. Original blots are presented in Fig. S4. (**D**, **E**) U2OS cells treated with 10 μM PP2 or 20 μM LY294002 in the presence of 2 μM etoposide as indicated for 3 days as indicated were subjected to SA-β-gal staining (**D**), and EdU incorporation assay (**E**). (**D**) The percentage of SA-β-gal positive cells are shown. E The percentage of EdU positive cells are shown. (**F**, **G**) U2OS cells transfected with p3XFLAG-CMV-14-Nectin-4 and cultured for the indicated times were subjected to immunoblot analysis. The phosphorylated protein levels of SFK (**F**) and Akt (**G**) normalized to their corresponding total protein levels were quantified using NIH ImageJ software. Representative immunoblot images from replicate experiments (n = 3, left panels) and the results of their statistical analysis (right panels) are shown. (**H**) U2OS cells transfected with p3XFLAG-CMV-14-Nectin-4, and treated with 10 μM PP2 or 20 μM LY294002 as indicated for 5 days were subjected to cell area measurement by microscopic image analysis. The cells were stained with Hoechst (blue), phalloidin (green), and anti-Flag antibody (magenta), respectively. Representative microscopic images (left panel) and dot plots of cell area between 5 and 95th percentiles (right panel) are shown as described in (**A**). Bar, 100 μm. The reduced-scale images are presented in Fig. S3. (**I**) U2OS cells transfected with siRNA for *Nectin-4* and treated with 10 μM PP2 or 20 μM LY294002 in the presence of 2 μM etoposide as indicated for 3 days were subjected to cell area measurement by microscopic image analysis after staining with crystal violet as described in (**A**). (**J**) Hs68 cells treated with 10 μM PP2 or 20 μM LY294002 in the presence of 0.5 μM etoposide as indicated for 3 days were subjected to cell area measurement by microscopic image analysis after staining with crystal violet as described in (**A**). (**K**) Hs68 cells were transfected with pcDNA3-HA containing oncogenic RasG12V, and treated with 10 μM PP2 or 20 μM LY294002 as indicated for 5 days were subjected to cell area measurement by microscopic image analysis. After incubation for 5 days, the cells were stained with Hoechst, phalloidin, and anti-HA antibody, respectively. Dot plots of cell area between 5 and 95th percentiles are shown as described in (**A**). Data are mean ± s.d. (n = 3 independent cultures). Statistical significance is shown using the Student’s *t*-test analysis; **p < 0.01; *p < 0.05.

The Nectin family proteins are known to recruit and activate SFK upon trans-interaction of Nectins^31, 32^, and Nectin-4 has been reported to activate PI3K^33, 34^ which can act as a downstream effector of SFK^35, 36^. Therefore, we examined whether SFK and PI3K are involved in etoposide-induced cellular senescence and cell size enlargement. The activation of SFK and PI3K was assessed by the phosphorylation levels of SFK at Tyr418 which is necessary for full catalytic activity of SFK^37^ and Akt at Ser473 which is a major phosphorylation site on PI3K downstream signaling^38^, respectively. The treatment of U2OS cells with a sub-lethal dose of etoposide induced cell size enlargement (Fig. 3A) accompanying the activation of SFK and PI3K (Fig. 3B). The pharmacological inhibition of SFK by PP2 inhibited not only the activation of SFK and Akt (Fig. 3B) but also cell size enlargement (Fig. 3A). Furthermore, the inhibition of PI3K by LY294002 (Fig. 3C) inhibited cell size enlargement (Fig. 3A). These results suggested that SFK and PI3K contribute to the regulation of cell size enlargement of etoposide-induce senescent cells. At this stage, however, it is also possible that the inhibition of SFK and PI3K directly suppressed senescence induction and consequently results in the prevention of cell size enlargement (*i.e.* SFK and PI3K might not play a direct role in the cell size regulation itself.). Therefore, to investigate whether SFK and PI3K are *bona fide* mediators of senescence-associated cell size enlargement or not, we tested the effect of PP2 and LY294002 treatment on senescence induction (Fig. 3D,E). The extent of etoposide-induced senescence was not affected by the PP2 and LY294002 treatment, as judged by SA-β-gal (Fig. 3D) and EdU proliferation assays (Fig. 3E). These results collectively indicate that SFK-PI3K pathway directly mediates the senescence-associated cell size enlargement rather than regulates senescence itself.

We next set out to examined whether SFK and PI3K are involved in Nectin-4 regulated cell size enlargement. After overexpression of Nectin-4, both SFK and PI3K were gradually activated (Fig. 3F,G). Furthermore, the treatment of PP2 and LY294002 remarkably inhibited the cell size enlargement induced by Nectin-4 overexpression (Fig. 3H). These results suggested that SFK and PI3K function as mediators of Nectin-4 regulated cell size enlargement.

As described above, SFK and PI3K contribute to cell size enlargement induced by etoposide treatment and Nectin-4 overexpression. However, it is not clear whether Nectin-4, SFK, and PI3K lie on the same signaling cascade regulating cell size enlargement in senescent cells. Therefore, we tested the effect of *Nectin-4* knockdown and the treatment of PP2 and LY294002 in the etoposide-induced cell size enlargement (Fig. 3I). Etoposide-induced cell size enlargement was effectively suppressed by *Nectin-4* knockdown, PP2, and LY294002, respectively, and additional treatment with PP2 and LY294002 to *Nectin-4* knockdown did not have an additive cell area suppression effect. These results suggested that Nectin-4, SFK, and PI3K lie on the same signaling cascade regulating cell size enlargement. Furthermore, to evaluate the generality of the contribution of SFK and PI3K in cell size enlargement during senescence, we used normal human fibroblast Hs68 cells (Fig. 3J,K). As a result, impairment of etoposide-induced cell area increase with PP2 and LY294002 was confirmed, and the combined treatment with PP2 and LY294002 had no significant synergistic effect on cell size increase (Fig. 3J). Moreover, cell spreading by oncogenic Ras–induced senescence of Hs68 cells was also impaired by PP2 and LY294002 treatment (Fig. 3K). Collectively, these results suggested that Nectin-4 activates the SFK-PI3K signaling pathway to induce the senescence-associated cell size enlargement.

### Nectin-4-induced cell size enlargement is mediated through Rac1 activity

**Figure 4:**
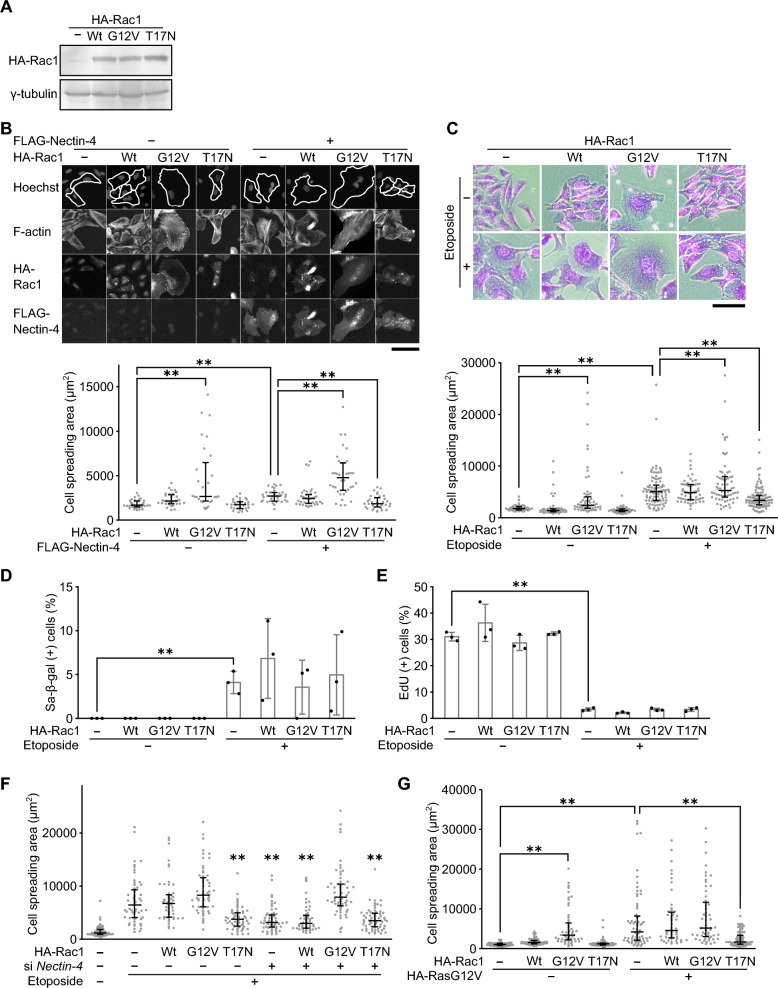
Nectin-4-induced cell size enlargement is mediated through Rac1 activity. (**A**) U2OS cells transfected with pcDNA3-HA-Rac1 containing Wt or the indicated mutations (G12V and T17N) were subjected to immunoblot analysis. Original blots are presented in Fig. S4. (**B**) U2OS cells transfected with pcDNA3-HA-Rac1 containing Wt, G12V, and T17N in combination with p3XFLAG-CMV-14-Nectin-4 as indicated, and cultured for a total of 5 days were subjected to cell area measurement by microscopic image analysis. Cells were stained with Hoechst, phalloidin, anti-HA antibody, and anti- FLAG antibody, respectively. White lines show cell edges determined from F-actin. Representative microscopic images (upper panel) and dot plots of cell area between 5 and 95th percentiles (lower panel) are shown. Upper and lower error bars (whiskers) and lines across the whiskers indicate the 75th and 25th percentiles and the median, respectively. Bar, 100 μm. The reduced-scale images are presented in Fig. S3. (**C**) U2OS cells transfected with pcDNA3-HA-Rac1 containing Wt, G12V, and T17N, selected with 800 μg/mL G418, and treated with 2 μM etoposide for 3 days were subjected to cell area measurement by microscopic image analysis after staining with crystal violet. Representative microscopic images (upper panel) and dot plots of cell area between 5 and 95th percentiles (lower panel) are shown as described in (**B**). Bar, 200 μm. The reduced-scale images are presented in Fig. S3. (**D**, **E**) U2OS cells transfected with pcDNA3-HA-Rac1 containing Wt, G12V, and T17N, selected with 800 μg/mL G418, and treated with 2 μM etoposide for 3 days were subjected to SA-β-gal (**D**) and EdU incorporation (**E**) assays. (**D**) The percentage of SA-β-gal positive cells are shown. (**E**) The percentage of EdU positive cells are shown. (**F**) U2OS cells transfected with siRNA for *Nectin-4* and overexpressed with pcDNA3-HA-Rac1 containing Wt, G12V, and T17N and treated with 2 μM etoposide as indicated for 5 days were subjected to cell area measurement by microscopic image analysis. Cells were stained with Hoechst, phalloidin, and anti-HA antibody, respectively. Dot plots of cell area between 5 and 95th percentiles are shown as described in (**B**). (**G**) U2OS cells transfected with pcDNA3-HA-Rac1 containing Wt, G12V, and T17N in combination with pcDNA3-HA containing oncogenic RasG12V as indicated, and cultured for a total of 5 days were subjected to cell area measurement by microscopic image analysis. Cells were stained with Hoechst, phalloidin, and anti-HA antibody, respectively. Dot plots of cell area between 5 and 95th percentiles are shown as described in (**B**). Data are mean ± s.d. (n = 3 independent cultures). Statistical significance is shown using the Student’s *t*-test analysis; **p < 0.01.

We next sought to delineate how the SFK-PI3K axis regulates the cell size enlargement. Nectin-4 has been reported to activate small GTPase Rac1 through the activation of PI3K in the context of carcinogenesis or tumor progression^34, 39^, and Rac1 is well known to be involved in multiple cellular processes including cell morphology^23, 24^. Therefore, we addressed whether Rac1 contributes to the Nectin-4-induced cell size enlargement. To this end, we constructed Rac1 mutants (G12V constitutive active and T17N dominant-negative forms) (Fig. 4A)^40, 41^. Overexpression of Wt-Rac1 slightly induced cell size enlargement (Fig. 4B), as previously reported^25, 26^. Furthermore, a constitutive active form of Rac1 (G12V) showed more potent effect on the cell size enlargement, while dominant-negative Rac1 (T17N) had no significant impact on cell size (Fig. 4B), indicating that cell size was increased in a Rac1 activity-dependent manner. Most importantly, when co-expressed with Nectin-4 and Rac1 mutants, the Nectin-4-induced cell size enlargement was unchanged and enhanced by Wt- and active G12V-Rac1, respectively, but significantly attenuated by dominant-negative T17N-Rac1 (Fig. 4B), leading us to speculate that the Rac1 activity is required for the Nectin-4-induced cell size enlargement. Consistent with this speculation, the etoposide-induced cell enlargement was also suppressed by overexpression of T17N-Rac1 (Fig. 4C). These results suggest that Rac1 acts downstream of Nectin-4 in the senescence-associated cell size enlargement. Furthermore, each Rac1 mutant did not affect the senescence induction itself, as evidenced by SA-β-gal and EdU proliferation assays (Fig. 4D,E), ruling out the possibility of direct effect of Rac1 activity on senescence induction. Next, we examined the relationship between Nectin-4 and Rac1 in etoposide-induced cell size enlargement. While both *Nectin-4* knockdown and T17N-Rac1 overexpression effectively suppressed etoposide-induced cell size enlargement, the combined treatment did not have an additive cell area suppression effect (Fig. 4F), suggesting that the Nectin-4 pathway and the Rac1 pathways are on the same cascade. Moreover, cell spreading by oncogenic Ras–induced senescence was also impaired by overexpression of T17N-Rac1 (Fig. 4G). In conclusion, these results indicate that Nectin-4 plays a causative role in the senescence-associated cell size enlargement mediated through the activation of SFK-PI3K-Rac1 axis.

### Nectin-4-induced cell size enlargement promotes senescent cell survival

**Figure 5:**
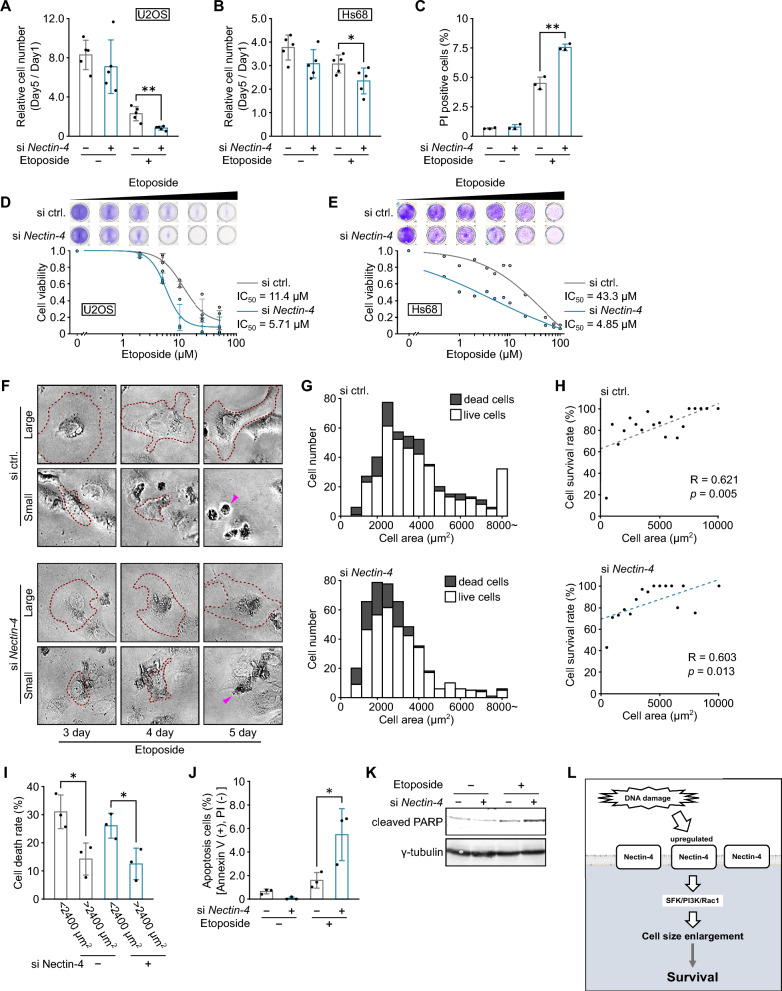
Nectin-4-induced cell size enlargement promotes senescent cell survival. (**A**, **B**) U2OS (**A**) and Hs68 (**B**) cells were transfected with siRNA for *Nectin-4* and treated with 2 µM or 0.5 μM etoposide for 5 days, and relative cell number was evaluated using WST-8 assay. (**C**) Death rate of U2OS cells treated as in (**A**, **B**) was determined by propidium iodide (PI) staining and flow cytometry. (**D**, **E**) U2OS (**D**) and Hs68 (**E**) cells were treated with different concentrations of etoposide, and cell viability was determined by crystal violet staining. The IC50 value was determined by fitting a sigmoidal dose–response curve to the data and shown in the graphs. (**F**–**I**) U2OS cells were transfected with siRNA for *Nectin-4* and treated with 2 µM etoposide for 5 days. Time-lapse images were acquired from day 3 to day 5 of etoposide treatment. Cell areas were measured at day 3, and cell survival rate was measured by tracking individual cells. Representative microscopic images (**F**), histograms (**G**), and scatter plots of survival rate and cell area (**H**) are shown. Dotted lines indicate cell outlines, arrow heads indicate dead cells. (**I**) Cell death rate was compared between cells with area of < 2400 µm^2^ and area of > 2400 µm^2^, which is the median area of *Nectin-4* knockdown cell. (**J**, **K**) U2OS cells transfected with siRNA for *Nectin-4* and treated with 2 µM etoposide for 7 days were subjected to annexin V/PI staining (**J**) and immunoblot analysis with an antibody to cleaved PARP, an indicator of apoptosis (**K**). Original blots are presented in Fig. S4. Statistical significance is shown using the Student’s *t*-test analysis; **p < 0.01; *p < 0.05. (**L**) Schematic illustration of this study indicating the enlargement of senescent cells mediated through Nectin-4. DNA damage induces the upregulation of Nectin-4 expression, which subsequently leads to the enlargement of cell area through the SFK/PI3K/Rac1 pathway, thereby contributing to cell survival.

Next, we investigated the functional significance of cell size enlargement in senescence. It has been reported that cell death is induced when the cell area is artificially reduced by culturing cells on the extracellular matrix-coated adhesive islands of defined size^42^, leading us to speculate that the survival of senescent cells can be promoted by Nectin-4-induced enlargement of cell size. To examine whether the suppression of cell size enlargement by *Nectin-4* knockdown leads to cell death, we compared cell numbers between *Nectin-4*-depleted and non-depleted senescent U2OS cells by using water-soluble tetrazolium salt-8 (WST-8) assay (Fig. 5A). The relative cell number (compared between before and after the 5-day treatment with etoposide) was markedly decreased by *Nectin-4* knockdown, while *Nectin-4* knockdown itself did not affect cell proliferation and survival in non-senescent cells (Fig. 5A). Similar results were obtained in the normal Hs68 cells (Fig. 5B). To directly assess cell death, *Nectin-4* depleted cells were stained with propidium iodide (PI), an indicator of cell death. *Nectin-4* knockdown significantly increased cell death when combined with etoposide treatment, suggesting that Nectin-4 can protect senescent cells against cell death (Fig. 5C). To investigate in more detail the role of Nectin-4 in senescent cell survival, we compared the sensitivity to etoposide (senescence-inducing stimulus) between *Nectin-4*-depleted and control cells (Fig. 5D,E). *Nectin-4* knockdown sensitized U2OS cells to etoposide as indicated by the significant decrease in IC50 (50% decrease) (Fig. 5D). The similar tendency was also observed in Hs68 cells (89% decrease in IC50) (Fig. 5E), supporting the speculation that Nectin-4-mediated size enlargement could contribute to the promotion of senescent cell survival. To directly determine the relationship between the cell size and survival, cell viability was compared between large and small cells by time-lapse imaging from day 3 to day 5 of etoposide treatment (Fig. 5F-I). Based on the acquired images, the cell area was measured on day 3, and the cells were classified as live or dead on day 5 (*i.e.,* cells survived until day 5 or died by day 5) (Fig. 5F). Frequency distribution histograms of cell areas of live and dead cells revealed that cells with a large area showed a tendency to survive until day 5, and conversely, smaller cells were more susceptible to cell death (Fig. 5G), implying that cell size enlargement contributes to cell survival. In line with this, there was a positive correlation between cell area and survival rate (R = 0.621 and 0.603 in *Nectin-4* intact and depleted cells, respectively) (Fig. 5H). Moreover, when cell death rate was compared between larger (> 2400 µm^2^, which is corresponding to the median of the dataset) and smaller cells (< 2400 µm^2^), smaller cells significantly showed a high death rate (Fig. 5I). Most importantly, the difference in survival rate between larger and smaller cells was equally observed in *Nectin-4*-depleted cells (Fig. 5G-I), which supports the direct relationship between cell size and survival capacity of senescent cells and rules out the involvement of other signaling pathways directly or indirectly regulated by Nectin-4 (*e.g.*, Akt-mediated cell survival pathway) in senescent cell survival. Finally, we should note here that cell size reduction was not an early sign of cell death (*e.g.,* cell shrinkage associated with cell death), because cell size decrease prior to death was hardly observed by continuous single cell tracking (Fig. S2). These results collectively suggest that senescent cell survival is enhanced by Nectin-4-mediated cell size enlargement, but not by Nectin-4-mediated survival signal pathway. Next, we examined whether the size reduction-induced cell death is caused by apoptosis, because it is known that apoptosis can be triggered by limiting cell adhesion^42^. The percentage of cells that were positive for Annexin V, a marker of apoptosis, was increased by the combined treatment with etoposide and *Nectin-4* knockdown (Fig. 5J). Furthermore, we also assessed the cleavage of PARP, a DNA repair protein that is cleaved by caspases during the execution phase of apoptosis. The PARP cleavage was increased by *Nectin-4* knockdown in senescent cells (Fig. 5K), suggesting that suppression of cell size enlargement sensitizes senescent cells to apoptosis. In sum, our results indicate that Nectin-4 promotes cell size enlargement in senescent cells by activating the SFK/PI3K/Rac1 pathway, which can contribute to senescent cell survival (Fig. 5L).

## Discussion

In the present study, we provided evidence that Nectin-4 has a causative role in senescence-associated cell size enlargement. Nectin-4 is an adhesion molecule that can regulate several cellular functions, such as cell movement, polarization, and differentiation^15, 16^, but its involvement in cell size regulation in senescence has not been described to date. We found that ectopic expression of Nectin-4 induced senescence-like enlarged cell morphology. Furthermore, the enlarged cell morphology induced by senescence-inducing stimuli was efficiently inhibited by siRNA-mediated depletion of *Nectin-4* in both tumor and normal cells. The cell area enlargement induced by replicative exhaustion was also impaired by *Nectin-4* knockdown, collectively indicating that Nectin-4 has a general function in the senescence-associated cell area enlargement. In contrast to cell area, cell volume was increased in senescent cells but not in cells overexpressing Nectin-4. At the same time, however, the senescence-associated volume increase was impaired by Nectin-4 knockdown. These results suggest that cell volume is also regulated by Nectin-4, but requires additional factor(s) that is activated in senescence, that is to say, Nectin-4 is sufficient to induce the cell area increase but requires additional factor(s) for the volume increase. Nonetheless, the mechanism governing the cell volume increase is considered to be also dependent on the Nectin-4-SFK-PI3K pathway, since the inhibition of SFK and PI3K attenuates the senescence-associated cell volume increase.

We further found that Nectin-4 induces the cell size enlargement mediated through the SFK-PI3K pathway. SFK has been shown to be recruited to and activated at Nectin-based cell–cell adhesion sites^31, 32^. On the other hand, PI3K is activated by Nectin-4 to increase survival and proliferation of cancer cells, although the underlying mechanism of Nectin-4-mediated PI3K activation is as yet unclear^33, 34^. However, SFK is known to directly activate PI3K by binding to a proline-rich region of the p85 regulatory subunit of PI3K^35^. In line with this, our results also showed that the SFK inhibition by PP2 attenuated the phosphorylation level of Akt, an indicator of PI3K activity. These results suggest in conjunction with the above studies that SFK activated by Nectin-4 in turn activates PI3K downstream of Nectin-4.

How the SFK-PI3K pathway contributes to the cell size enlargement? Several of the above-mentioned studies concerning SFK-PI3K signaling point out that the activation of SFK-PI3K cascade results in the sequential activation of Rac1 signaling pathway^31, 32, 34, 39^. Generally, in response to environmental cues, cell membrane receptors and adhesion molecules cooperatively regulate Rac1 activity through GEFs and GTPase activating proteins. It is well known that PI3K activates Rac1 by stimulation of Rac1-GEF Tiam1^43^. In the GTP-bound active state, Rac1 binds to a large number of effector molecules that ultimately result in the stimulation of signaling cascades regulating general cellular responses, such as cell spreading, cytoskeletal change, microtubule dynamics, cell polarity, etc. Consistent with these known functions of Rac1, we observed that ectopic expression of the dominant-negative form of Rac1 completely abolished the senescence-associated cell enlargement. Furthermore, the dominant-negative Rac1 also abrogated the cell size enlargement induced by Nectin-4 overexpression, indicating that Rac1 is a vital element for the senescence-associated cell size enlargement induced by the Nectin-4-SFK-PI3K axis.

It has been reported that integrins binding to the extracellular matrix contributed to survival by adhesion signal^44^, and that external restriction of cell regions caused genetic changes such as cell adhesion factors^45^. In the present study, we showed that the cell size enlargement by Nectin-4 leads to survival. Therefore, this cell survival may be due to the elevation adhesion signal accompanying the increase the cell size enlargement. Although the effect of two-dimensional spreading of the cellular area in a three-dimensional environment is unclear, it was reported that senescent cells show large cell area in tissue^27^. Furthermore, the levels of stress fiber and vinculin increase in correlation with an increase in the surface area of 3D cultures^46^. Therefore, it is considered that a cell area spreading increases the adhesion area and leads to survival promotion even in three dimensions.

Recently, several studies have shown that the persistent presence of senescent cells is a significant driver of numerous age-related diseases, including the development of cancer metastases^47, 48^. These results may suggest that the vital link between senescence and cancer. Nectin-4 has been reported to be differentially expressed in various types of cancers such as bladder, breast, ovarian, and lung cancers, and its expression is associated with poor cancer prognosis^39, 49, 50^. By virtue of its differential expression, an antibody–drug conjugate targeting Nectin-4 (enfortumab vedotin) that delivers an anticancer agent to Nectin-4-expressing tumors has been developed for the treatment of bladder cancer patients^49, 51^. Given that misregulation of cell size and shape is a histological hallmark of a wide range of malignant lesions and correlates with tumorigenicity^52^, Nectin-4 can also contribute to malignancy through the regulation of cancer cell size. However, so far, the molecular mechanism of how Nectin-4 is involved in the poor prognosis of cancer is not clear, and thus our study also warrants further investigations to elucidate the relationships among Nectin-4, cell size, and cancer malignancy. Regardless of their outcomes, our results clearly show that Nectin-4 is responsible for the senescence-associated cell size enlargement through the activation of SFK-PI3K-Rac1 pathway, which may shed new light on the Nectin-4 roles in senescence.

## Methods

### Cell culture, treatment, and transfection

U2OS cells (a human osteosarcoma line; ATCC, Rockville, MD, USA) and Hs68 (normal human diploid fibroblasts; IFO50350; JCRB Cell Bank, Osaka, Japan) cells were cultured in DMEM (Wako, Osaka, Japan) supplemented with 10% fetal bovine serum. The cells were treated with etoposide (Sigma Aldrich, St Louis, MO, USA) to induce DNA double-strand breaks. For senescence induction, U2OS and Hs68 cells were treated with 2 and 0.5 μM etoposide (Sigma Aldrich), respectively, for 48 h and cultured in the medium without the drug for additional 5 days to develop senescent phenotypes^18–20, 30^. Transfection with expression vectors was conducted by using Effectene Transfection Reagent (Qiagen, Venlo, Netherlands) according to the manufacturer’s instruction. Where indicated, the transfected cells were selected by 800 µg/mL G418 (Wako) for 5 days. PP2 (Merck Millipore) and LY294002 (Merck Millipore) were used to inhibit SFK and PI3K, respectively. Stock solutions of etoposide, PP2, and LY294002 were prepared in dimethyl sulfoxide.

### Plasmid constructions

For the construction of pcDNA3-HA-Nectin-4, an expression vector for N-terminal HA-tagged human full-length Nectin-4 (NCBI Reference Sequence: NM_030916.3), the Nectin-4 cDNA was amplified with a pair of primers (a forward primer: 5′-CGTGGATCGAATTCATGCCCCTGTCCCTGGGAGCCGAGAT-3′ and a reverse primer: 5′-CGTGCTCGGCGGCCGCTCAGACCAGGTGTCCCCGCCCATT-3′) using a cDNA sample prepared from U2OS cells as a template. The resultant fragment was digested with Eco RI and Not I, and cloned into pcDNA3 vector (Invitrogen, Carlsbad, CA, USA). To generate p3XFLAG-CMV-14-Nectin-4, an expression vector for C-terminal Flag-tagged Nectin-4, the fragment of Nectin-4 was amplified with the same forward primer and a reverse primer 5′-GCGGTACCGTGACCAGGTGTCCCCGCCCATTGATGT-3′ using pcDNA3-HA-Nectin-4 as the template and cloned into the Eco RI-Kpn I site of p3XFLAG-CMV-14 vector (Sigma-Aldrich). To construct the expression vector, pcDNA3-HA-Rac1-Wt that contains full-length human Rac1-Wt (NCBI Reference Sequence: NM_006908.5), the corresponding cDNA fragments were amplified with a pair of primers (a forward primer: 5′-GCGAATTCACCATGCAGGCCATCAAGTGTG-3′ and a reverse primer: 5′-GCGAATTCTTACAACAGCAGGCATTTTCTC-3′). The resultant fragments were digested with Eco RI, and cloned into downstream of the HA tag sequence in the pcDNA3-HA vector. For the construction of Rac1 mutants (G12V and T17N), PCRs were performed using mutagenic primers (a forward primer: 5′-GCGGATTCACCATGCAGGCCATCAAGTGT-3′ and a reverse primer: 5′-GTGGTGGTGGGAGACGTAGCTGTAGGTAAA-3′ for G12V; a forward primer: 5′-GCGGATTCACCATGCAGGCCATCAAGTGT-3′ and a reverse primer: 5′-GTGGTGGTGGGAGACGGAGCTGTAGGTAAAAATTGCCTACTG-3′ for T17N) with pcDNA3-HA-Rac1 as the template to generate pcDNA3-HA-Rac1-G12V and pcDNA3-HA-Rac1-T17N. To construct pcDNA3-HA-RasG12V, an expression vector that contain full-length human G12V-H-Ras, the human RasG12V cDNA was amplified with a pair of primers (a forward primer: 5′-GCGAATTCATGACGGAATATAAGCTGGTG-3′ and a reverse primer: 5′-GCAGCGGCCGCTCAGGAGAGCACACACTTG-3′) using a vector, pCMV-HA-RasG12V (kindly provided by Dr. K Kaibuchi, Nagoya University, Japan), as a template. The resulting fragment was digested with Eco RI and Not I and cloned into downstream of the HA tag sequence in the pcDNA3-HA vector (Invitrogen).

### Immunoblot analysis

The cells were lysed in lysis buffer (1% Triton X-100, 20 mM Tris–HCl [pH 7.5], 1 mM EDTA, 1 mM EGTA, 150 mM NaCl, 10 mM 2-mercaptoethanol, 5 μg/ml leupeptin, 1 mM APMSF, 1 mM Na_3_VO_4_), and the lysates were separated by SDS–polyacrylamide gel electrophoresis and blotted onto Immobilon polyvinylidene difluoride membrane (Merck Millipore). Each protein was detected using primary antibodies as indicated, AP-conjugated secondary antibodies, and the chromogenic NBT/BCIP (Nacalai Tesque, Kyoto, Japan) substrates.

### Antibodies

Anti-FLAG M2 monoclonal antibody (F3165) and anti-γ-tubulin antibody (T6557) were obtained from Sigma Aldrich; anti-P-Akt (S473) antibody (#9271S), anti-Akt antibody (#9272S), and cleaved PARP (Asp214) antibody (#9541) were from Cell Signaling Technology (Beverly, MA, USA); AP-conjugated anti-mouse antibody (S372B) and AP-conjugated anti-rabbit antibody (S373B) were from Promega; anti-Nectin-4 antibody (ab192033) was from Abcam; anti-P-SFK (Y418) antibody (44-660G) was from ThermoFisher Scientific (Waltham, MA, USA); anti-SFK antibody (OP07) was from Merck Millipore; anti-HA 3F10 monoclonal antibody (1867423) was from Roche (Basel, Switzerland); AP-conjugated anti-rat antibody (sc-2021) was from Santa Cruz Biotechnology (Santa Cruz, CA, USA).

### Immunofluorescence

For immunofluorescence analysis, the cells were fixed with 3.7% formaldehyde and permeabilized in 0.5% Triton X-100. The fixed cells were incubated with primary antibodies overnight at 4 °C followed by incubation with the Alexa Fluor 488-conjugated secondary antibody (Life Technologies) for 1 h at room temperature. After staining cell nuclei with 10 μM Hoechst 33,258 and actin filaments with FITC-conjugated phalloidin (1:250, A12379, Thermo Fisher Scientific) where indicated, the cells were observed under fluorescence microscope (model BZ-9000; Keyence).

### Senescence assay

To measure SA-β-gal activity, Senescence β-Galactosidase staining kit (Cell Signaling Technology) was used according to the manufacturer’s instructions. Briefly, the cells were fixed with 2% formaldehyde/0.2% glutaraldehyde for 15 min and incubated with SA-β-Gal staining solution (1 mg/ml 5- bromo-4-chloro-3-indolyl-β-D-galactoside, 40 mM citric acid/sodium phosphate [pH 6.0], 5 mM potassium ferrocyanide, 5 mM potassium ferricyanide, 150 mM NaCl, 2 mM MgCl_2_) for 24 h at 37 °C. The stained cells were examined under fluorescence microscope (model BZ-8000; Keyence, Osaka, Japan). Senescent cells were identified as blue-stained cells. For EdU incorporation proliferation assay, the cells were labeled with EdU for 3 h prior to fixation, and then EdU incorporation was visualized by using Click-iT EdU Imaging Kit (Life Technologies) according to the manufacturer's instructions. The stained cells were observed under fluorescence microscope (model BZ-9000; Keyence). At least 100 cells in randomly selected microscopic fields were counted to determine the percentage of SA-β-gal positive cells and EdU positive cells.

### Apoptosis assay

The U2OS cells were harvested using trypsin and then resuspended in binding buffer (#556547, BD Biosciences). The apoptosis assay was performed by double staining the cells with 1 μL of Annexin V-FITC and 2 μL of PI (#556,547, BD Biosciences), followed by incubation for 15 min at room temperature in the dark. Subsequently, the cells were observed under a fluorescence microscope (model BZ-9000; Keyence). Apoptotic cells were identified as Annexin V-FITC-positive and PI-negative cells. To determine the percentage of Annexin V-positive cells and PI-negative cells, at least 80 cells were counted in randomly selected microscopic fields.

### RNA isolation and quantitative PCR (qPCR)

Total RNA was isolated from siRNA-transfected U2OS cells using RNeasy Mini Kit (QIAGEN) according to the manufacturer’s instruction. cDNA was synthesized using ReverTra Ace qPCR RT Master Mix with gDNA Remover (TOYOBO), and the resulting cDNAs were subjected to qPCR (LightCycler480 Real-Time PCR System; Roche Applied Science) using specific primers for GAPDH (a forward primer: 5′-CAATGACCCCTTCATTGACCT-3′ and a reverse primer: 5′-ATGACAAGCTTCCCGTTCTC-3′), Nectin-4 (a forward primer: 5′-CTGCCATGTCAGCAATGAGT-3′ and a reverse primer: 5′-TGGAATGCTGATGACTTGGAG-3 ‘), p21 (a forward primer: 5′-CGACTGTGATGCGCTAATG-3′ and a reverse primer: 5′-TCTCGGTGACAAAGTCGAAG-3′). Relative expression levels were calculated by the 2^(ΔΔCt) method (ΔCt sample-ΔCt calibrator).

### RNA interference

ON-TARGETplus Smart Pool siRNA for *Nectin-4* (L-004301–00) and its control siRNA (D-001810–10) were from Dharmacon Horizon Discovery (Lafayette, CO, USA). U2OS cells were seeded and transfected with 30 nM siRNA using Lipofectamin RNAiMAX Transfection Reagent (ThermoFisher Scientific) according to the manufacturer’s instructions.

### Cell spreading area measurement

For cell size measurement, the cells were stained with crystal violet (Wako) or subjected to immunofluorescence staining as described above. The purple stained area (for crystal violet staining) or the phalloidin stained area (for immunofluorescence) was identified as the cell spreading area and measured by using NIH ImageJ software.

### Cell volume measurement

To measure cell volume, the cells were trypsinized and resuspended in phosphate-buffered saline, and the cell volume was quantified using a Scepter Handheld automated cell counter with 60 μm Scepter Sensors and Scepter software Pro 2.1. (Millipore), which employs the Colter principle of impedance-based particle detection.

### Flow cytometry

To analyze cell death, the cells were stained with 1 µL/mL PI (Sigma-Aldrich, P4864) for 15 min, and dead cells were determined by measuring the PI fluorescence intensity using BD Accuri C6 Plus (BD Biosciences). At least ten thousand events were analyzed for each sample and the data were plotted using BD Accuri C6 Plus software (BD Biosciences). To measure cell volume, the cells were trypsinized and resuspended in phosphate-buffered saline, and the cell volume was measured based on forward scatter (FSC) by using BD Accuri C6 Plus (BD Biosciences). At least ten thousand events were analyzed for each sample and the data were plotted using BD Accuri C6 Plus software (BD Biosciences).

### WST-8 assay

To assess the proliferation rate, U2OS cells or Hs68 were cultured at a concentration of approximately 1 × 10^4^ cells per well in 96-well plates. The cells were treated with different conditions and then incubated with WST-8 solution according to the manufacturer’s instructions. The absorbance was measured at 450 nm with a spectrophotometer.

### Statistical analysis

The two-tailed Student’s *t*-test was used to calculate *p*-values for all datasets.

### Supplementary Information

Supplementary Information.

## Data Availability

The datasets used and/or analysed during the current study available from the corresponding author on reasonable request.
