# Supplementary material for: Nectin-4 regulates cellular senescence-associated enlargement of cell size
Source: Sci Rep. 2023 Dec 7;13:21602. doi: 10.1038/s41598-023-48890-z (PMC10703872; doi:10.1038/s41598-023-48890-z)
Supplement: Supplementary file 1 — Supplementary Information. [file 41598_2023_48890_MOESM1_ESM.pdf]

**Supplementary Information for**

# Nectin-4 regulates cellular senescence-associated enlargement of cell size

Ryoko Katasho[1], Taiki Nagano[2], Tetsushi Iwasaki[1,2], and Shinji Kamada[1,2,*]

[1] Department of Biology, Graduate School of Science, Kobe University, 1-1 Rokkodai-cho, Nada-ku, Kobe 657-8501, Japan.

[2] Biosignal Research Center, Kobe University, 1-1 Rokkodai-cho, Nada-ku, Kobe 657-8501, Japan.

*Correspondence: skamada@kobe-u.ac.jp

## Figure S1

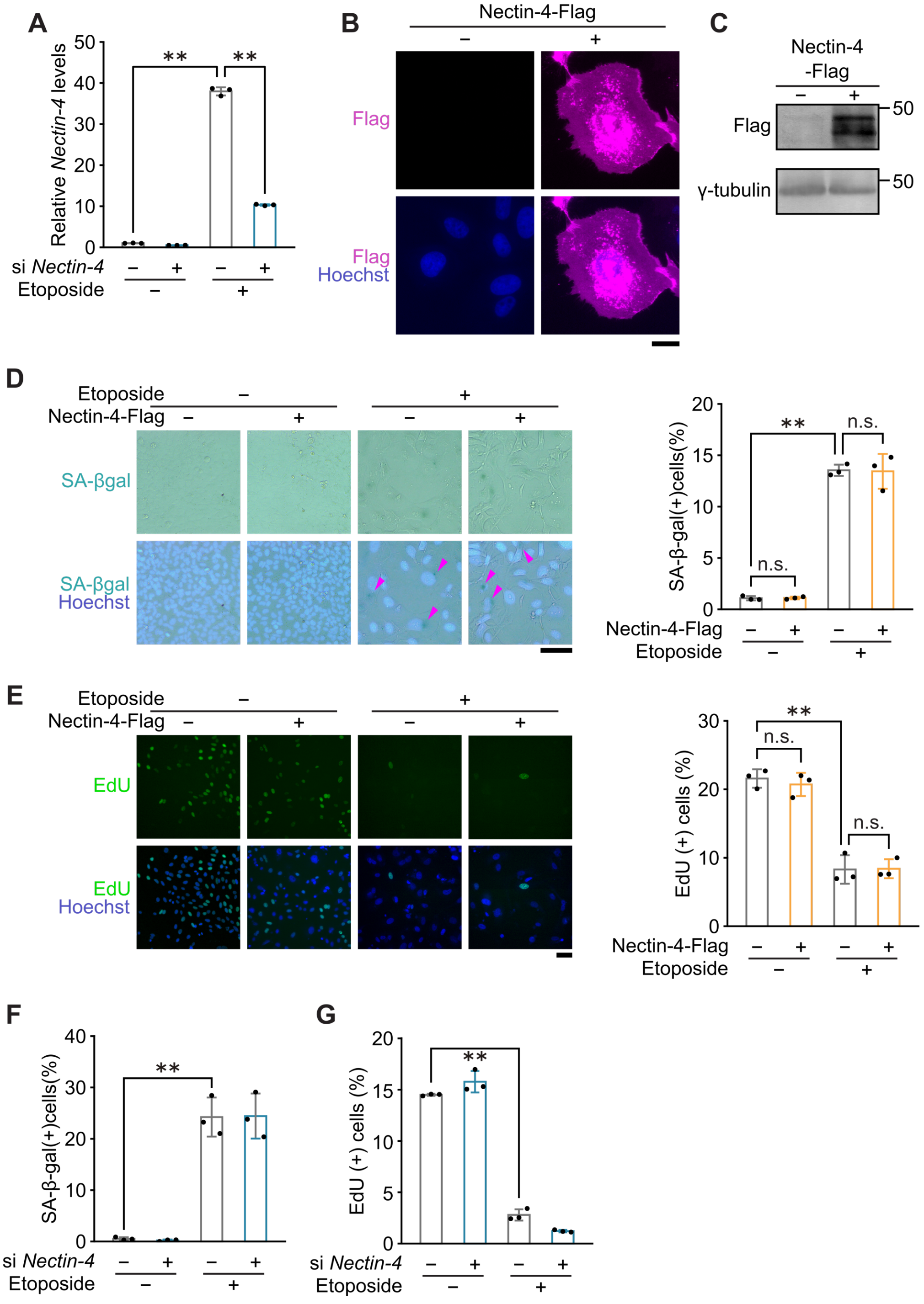


**Figure S1.　Nectin-4 is not involved in DNA damage-induced senescence itself.**

(A) U2OS cells transfected with siRNA for *Nectin-4* and treated with 2 μM etoposide for 7 days were subjected to qPCR analysis.　Data are mean ± s.d. (n = 3 independent cultures).

(B, C) U2OS cells transfected with p3XFLAG-CMV-14-Nectin-4 were subjected to immunofluorescence (B) and immunoblot (C) analyses. The cells were stained with Hoechst (blue) and anti-Flag antibody (magenta), respectively (B). Bar, 50 μm. Original blots are presented in Fig. S4. (D, E) U2OS cells transfected with p3XFLAG-CMV-14-Nectin-4, selected with 800 μg/mL G418, and treated with 2 μM etoposide for 7 days were subjected to SA-β-gal (D) and EdU incorporation (E) assays. Representative microscopic images (D, E left panels) and the percentage of SA-β-gal positive cells (D right panel) and EdU positive cells (E right panel) are shown. Arrow heads indicate SA-β-gal positive cells (D). Green fluorescence represents the EdU-positive cells; blue fluorescence from the Hoechst stain represents the total cells. Bars, 200 μm (D) and 100 μm (E). (F, G) U2OS cells transfected with siRNA for *Nectin-4* and treated with 2 μM etoposide for 7 days were subjected to SA-β-gal (F) and EdU incorporation (G) assays. The percentage of SA-β-gal positive cells (F) and of EdU positive cells (G) are shown. Data are mean ± s.d. (n = 3 independent cultures). Statistical significance is shown using the Student's *t*-test analysis; $^{**}p < 0.01$; n.s., not significant ($p > 0.05$).

**Figure S2**

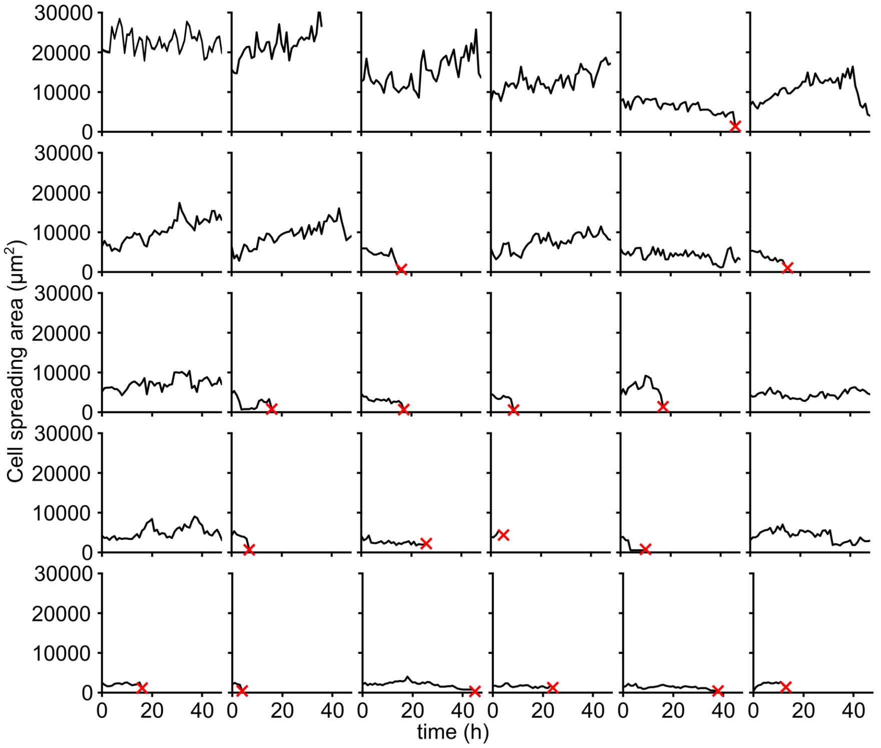


**Figure S2. Continuous single cell tracking of size and viability.**

U2OS cells were transfected with siRNA for *Nectin-4* and treated with 2 μM etoposide for 5 days. Time-lapse images were acquired from day 3 to day 5 of etoposide treatment. The results of single-cell tracking of cell spreading areas (Cells #1-#30) are shown in the graphs. The red cross marks indicate the point of cell death.

## Figure S3

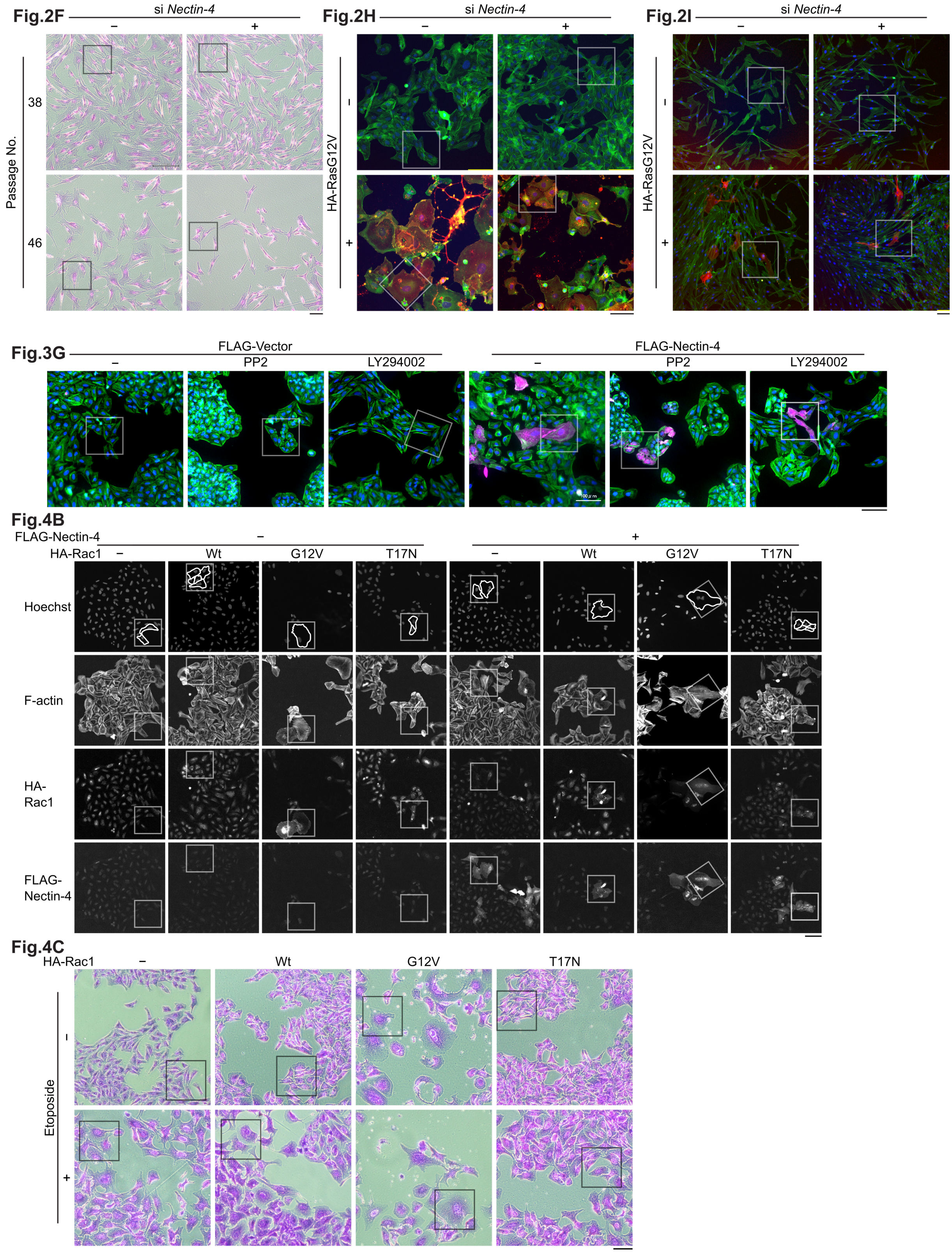
Fig.2F
si Nectin-4
–
+
Passage No.
38
46
Fig.2H
si Nectin-4
–
+
HA-RasG12V
–
+
Fig.2I
si Nectin-4
–
+
HA-RasG12V
–
+
Fig.3G
FLAG-Vector
–
PP2
LY294002
FLAG-Nectin-4
–
PP2
LY294002
Fig.4B
FLAG-Nectin-4
–
+
HA-Rac1
–
Wt
G12V
T17N
–
Wt
G12V
T17N
Hoechst
F-actin
HA-Rac1
FLAG-Nectin-4
Fig.4C
HA-Rac1
–
Wt
G12V
T17N
Etoposide
–
+

**Figure S3. Reduced-scale image.**

The reduced-scale images of each figure in the main text are shown. The squares represent the images used in the main text. Bars, 100 μm.

**Figure S4**

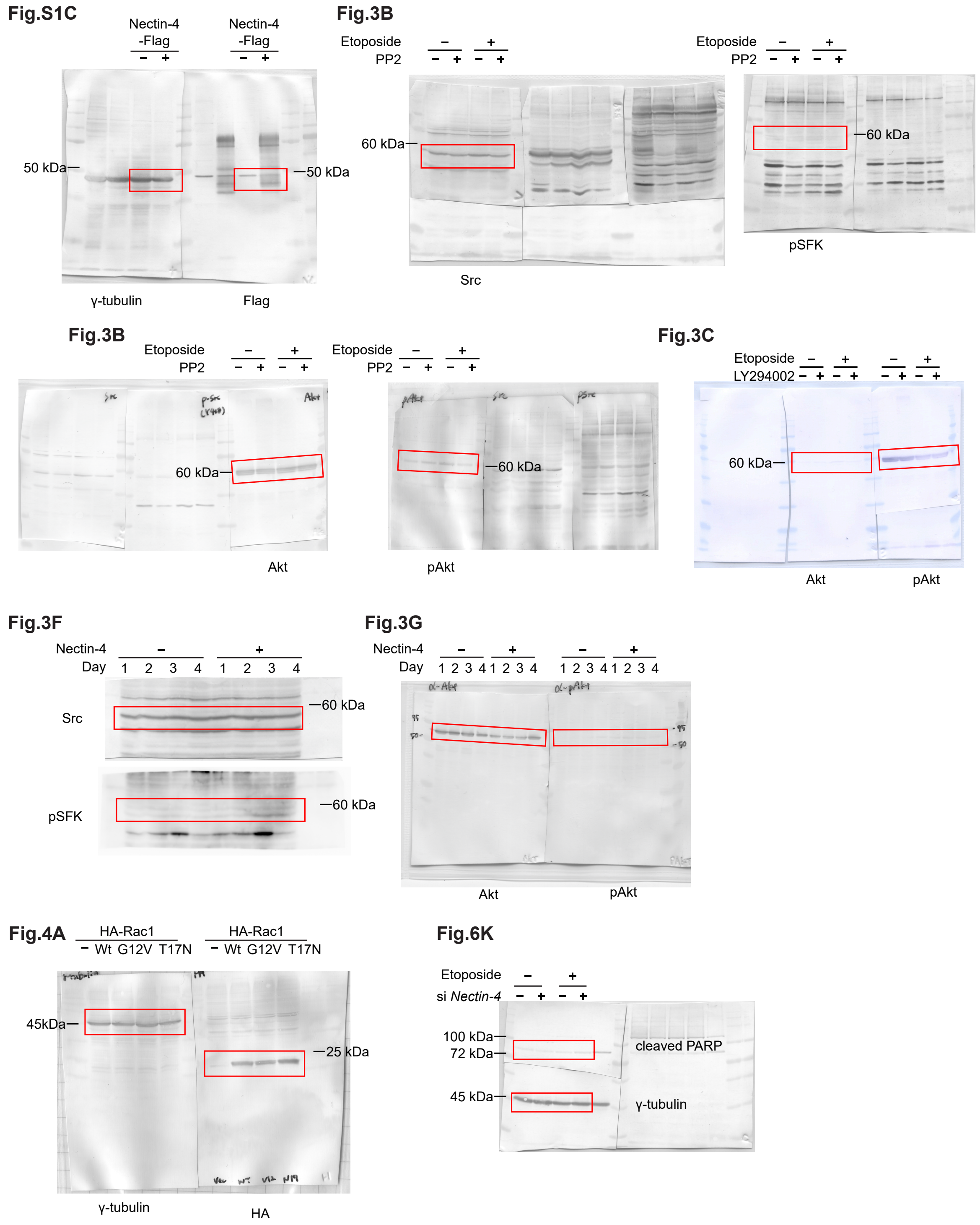

Fig.S1C
Nectin-4 -Flag
– +
Nectin-4 -Flag
– +
50 kDa
50 kDa
γ-tubulin
Flag
Fig.3B
Etoposide – +
PP2 – + – +
60 kDa
Src
Etoposide – +
PP2 – + – +
60 kDa
pSFK
Fig.3B
Etoposide – +
PP2 – + – +
60 kDa
Akt
Etoposide – +
PP2 – + – +
60 kDa
pAkt
Fig.3C
Etoposide – + – +
LY294002 – + – + – + – +
60 kDa
Akt
pAkt
Fig.3F
Nectin-4 – +
Day 1 2 3 4 1 2 3 4
Src
60 kDa
pSFK
60 kDa
Fig.3G
Nectin-4 – + – +
Day 1 2 3 4 1 2 3 4 1 2 3 4 1 2 3 4
Akt
pAkt
Fig.4A
HA-Rac1
– Wt G12V T17N
HA-Rac1
– Wt G12V T17N
45kDa
25 kDa
γ-tubulin
HA
Fig.6K
Etoposide – +
si Nectin-4 – + – +
100 kDa
72 kDa
45 kDa
cleaved PARP
γ-tubulin

**Figure S4. Full size immunoblots.**

Uncropped images of each blot are shown. Cropping lines are indicated with squares.
